# Dysfunctional Implantable Loop Recorder Post‐Electrical Cardioversion: A Report of Two Cases

**DOI:** 10.1002/ccr3.70740

**Published:** 2025-08-23

**Authors:** Wael Zaher, Lorenzo Marcon, Antonio Sorgente

**Affiliations:** ^1^ Department of Cardiology Centre Hospitalier EpiCURA Hornu Boussu Belgium

**Keywords:** cardiovascular implantable electronic device, electrical cardioversion, electromagnetic interference, implantable loop recorder

## Abstract

We report two occurrences of implantable loop recorder (ILR) dysfunction following electrical cardioversion, likely due to anterior–posterior patch positioning. Close proximity to the electrical field may affect ILRs, emphasizing the need for careful patch placement, similar to other cardiovascular implantable electronic devices.

**Abbreviations**
AF: atrial fibrillation
CIED: cardiovascular implantable electronic devices
ECV: electrical cardioversion
EMI: electromagnetic interference
ILR: implantable loop recorder

## 1 Introduction

Implantable loop recorders (ILRs) are commonly used for long‐term cardiac rhythm monitoring, especially in patients with unexplained syncope. Electrical cardioversion (ECV) is a regular intervention for atrial arrhythmia, including atrial fibrillation (AF). However, the interaction between ECV and ILR functionality is not well documented. Here, we report a case series of two occurrences of ILR dysfunction following ECV, highlighting the potential risks and the importance of appropriate patch positioning.

## 2 Case History

### 2.1 Case No. 1

**FIGURE 1:**
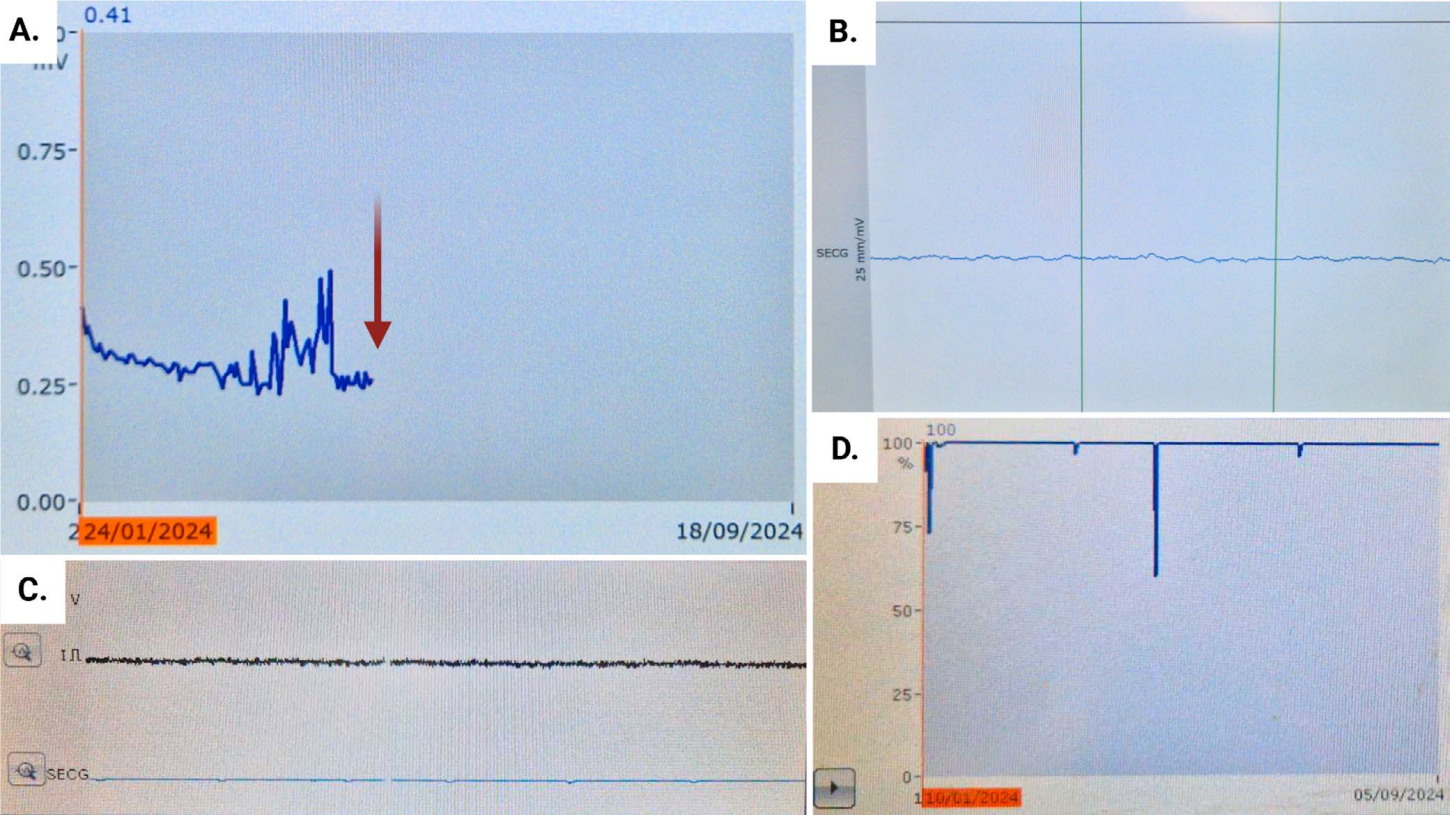
Central figure. (A) R wave detection tendency, with red arrow showing abrupt loss of detection from the day of ECV in patient 1. (B) Example of recording stored since the day of ECV in patient 1. (C) Example of subcutaneous electrocardiogram during interrogation in patient 2. (D) Interference tendency in patient 2, at 100% most of the time. ECV, electrical cardioversion.

A 72‐year‐old female, without known cardiac disease, underwent an ILR (Biomonitor IIIm, Biotronik Inc) implantation for recurrent unexplained syncope. Two months later, a coronary computed tomography angiography for chest pain revealed a significantly suspicious stenosis of the left anterior descending artery. Coronary angiography showed no significant coronary artery disease; however, AF developed during the procedure. ECV was performed using an anterior–posterior electrode patch position with a biphasic shock at 150 J, without complications, and the patient was discharged the same day. In the following days, remote monitoring indicated persistent abnormal artefactual data, which was confirmed one month later during a follow‐up consultation (Figure 1).

### 2.2 Case No. 2

A 69‐year‐old male underwent an electrophysiology study for syncope in the setting of heart failure with moderately reduced ejection fraction. The electrophysiology study was normal, and an ILR (Biomonitor IIIm, Biotronik Inc) was implanted. At follow‐up, AF was documented, becoming persistent and requiring an ECV 16 months post‐implantation. The ECV was performed with an anterior–posterior electrode patch position with a biphasic shock at 150 J, and was uneventful. In the days following the ECV, ILR interrogation showed loss of detection with abnormal artefactual data starting from the day of ECV.

## 3 Diagnosis and Follow‐Up

In both cases, chronology analysis revealed an abrupt loss of detection and interruption in data transmission on the day of ECV (Figure 1). We hypothesize that the anterior–posterior positioning of the ECV patches placed the ILR within the electrical field, leading to its dysfunction. In the first case, the decision was made to remove the ILR and to implant a new one. The removed device was sent to the manufacturer for analysis, but no relevant findings were obtained. In the second case, a concerted decision with the patient was made to leave the ILR in place, and follow‐up was uneventful.

## 4 Discussion

**FIGURE 2:**
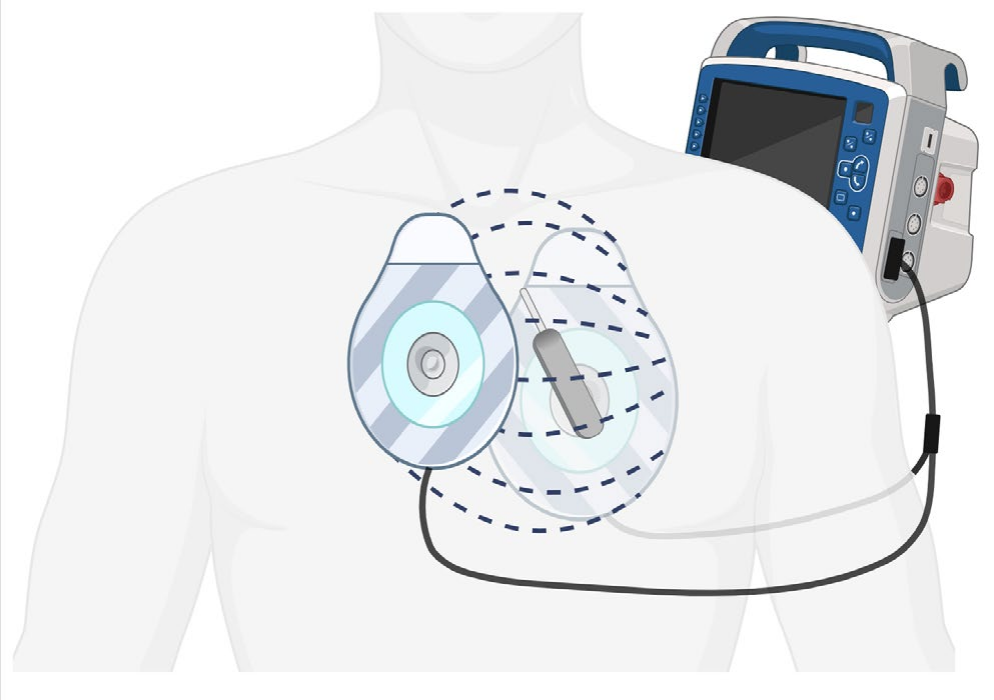
Patch positioning relative to the loop recorder. An anterior–posterior position patch configuration with a conventional pectoral subcutaneous ILR at a 45° angulation places the ILR in close proximity to the anterior patch and at the center of the electrical field. Created with BioRender.com. ILR, implantable loop recorder.

This case series reports unexpected ILR dysfunction following ECV. ECV involves delivering a high‐energy shock to the heart to restore sinus rhythm, and the patch placement can significantly influence the distribution of the electrical field. The anterior–posterior position is a commonly used configuration for ECV. However, in patients with implanted devices like ILRs, this configuration may pose risks. The ILR, typically implanted subcutaneously in the left pectoral region, may lie within the vector of the electrical field generated by anterior–posterior positioning (Figure 2). The high voltage and current density in this field can interfere with the electronic circuitry of the ILR, leading to malfunction or artefactual data recording. This electromagnetic interference (EMI) could manifest as a sudden cessation of accurate data transmission, as observed in our cases. Both reported cases involve the Biotronik Biomonitor IIIm. It remains uncertain whether other ILRs are also susceptible to this risk.

Previous studies have documented EMI‐related issues with cardiovascular implantable electronic devices (CIEDs), such as pacemakers and implantable cardioverter‐defibrillators (ICDs), during ECV. The extent of EMI generated by ECV depends on several factors, including the proximity of the device to the field, the energy of the shock, and the orientation of the device relative to the current flow. While ECV can be safely performed in patients with CIEDs, with a low rate of complications [1, 2] and minimal modifications to CIED parameters [3]. However, it may be associated with a higher risk of lead reintervention and generator replacement [4]. Although a 10‐cm distance between the anterior shock pad and the generator is commonly used to minimize malfunction risk, there are no specific recommendations on this topic in the latest ESC and ACC/AHA recommendations on atrial fibrillation [5, 6]. Additionally, as chest wall conformation influences the manifestation of AF [7], anatomical variations may affect the susceptibility of ILRs to electrical interference. Specifically, a narrow anteroposterior thoracic diameter could increase ILR exposure to electrical fields, potentially heightening the risk of dysfunction. Therefore, chest wall anatomy should be considered when managing patients, particularly regarding ILR implantation in AF patients who may undergo ECV.

To our knowledge, there are no reports specifically addressing ILR dysfunction post‐ECV. These two cases add to the limited body of evidence, suggesting that ILRs are similarly susceptible to EMI from ECV, particularly when positioned in the field's direct path, typically anterior–posterior for a conventional pectoral ILR. To mitigate this risk, anterolateral configuration may be considered to reduce the likelihood of the ILR being exposed to the full force of the electrical field. Minimizing current flow through the ILR is recommended to prevent potential damage during ECV [8]. Clinicians should be aware of these considerations and tailor their approach to minimize the potential for device interference.

## 5 Conclusion

These cases highlight the risk of ILR dysfunction following ECV with anterior–posterior patch placement and emphasize proper patch placement to avoid device dysfunction.

## Author Contributions

**Wael Zaher:** conceptualization, visualization, writing – original draft, writing – review and editing. **Lorenzo Marcon:** conceptualization, writing – review and editing. **Antonio Sorgente:** supervision, writing – review and editing.

## Disclosure

The authors have nothing to report.

## Consent

The authors confirm that written informed consent was obtained from both patients.

## Data Availability

Data supporting the findings of this report are included within the article; additional clinical details are available upon reasonable request from the corresponding author, subject to patient confidentiality and ethical approval.
